# OUT-OF-HOME MOBILITY, DAILY WORKING MEMORY, AND NEIGHBORHOOD WALKABILITY IN HEALTHY OLDER ADULTS

**DOI:** 10.1093/geroni/igad104.0907

**Published:** 2023-12-21

**Authors:** Minxia Luo, Eun-Kyeong Kim, Robert Weibel, Mike Martin, Christina Roecke

**Affiliations:** University of Zurich, Zurich, Zurich, Switzerland; Luxembourg Institute of Socio-Economic Research, Luxembourg, Diekirch, Luxembourg; University of Zurich, Zurich, Zurich, Switzerland; University of Zurich, Zurich, Zurich, Switzerland; University of Zurich, Zurich, Zurich, Switzerland

## Abstract

Out-of-home mobility could enhance cognitive performance, but environmental contexts — such as neighborhood walkability — may influence the associations. This study examined daily out-of-home mobility in relation to daily cognitive performance in healthy older adults and the moderator effects of neighborhood walkability therein. Participants wore a custom-built mobile GPS sensor („uTrail“) and completed smartphone-based working memory assessments for 15 days, and responded to the baseline Neighborhood Environment Walkability Scale. Analyses included 947 days’ data from 109 Swiss older adults aged 65 to 89 years. Out-of-home mobility (i.e., out-of-home time, maximum distance from home) were extracted from the GPS data. Multilevel models showed that daily out-of-home time and daily maximum distance from home were not associated with daily working memory performance across the study sample, but the associations were moderated by neighborhood walkability. Out-of-home time and maximum distance from home were positively (vs. negatively) associated with working memory performance in participants who lived in neighborhood with higher (vs. lower) street connectivity and more (vs. less) access to places for walking and cycling. Additionally, maximum distance from home were positively (vs. negatively) associated with working memory performance in participants in neighborhood with lower (vs. higher) mixture of land use. To conclude, spending more time out of home and travelling longer distance away from home could be beneficial for daily cognitive performance of older adults who lived in well-connected and higher-walkable and -bikeable neighborhood. Travelling longer distance could also benefit those who lived in a neighborhood with a lower mixture of land use.

